# Retraction: 2,5-Hexanedione induced apoptosis in rat spinal cord neurons and VSC4.1 cells via the proNGF/p75NTR and JNK pathways

**DOI:** 10.1042/BSR20204264_RET

**Published:** 2026-08-14

**Authors:** Mengxin Luo, Xiaoxia Shi, Qi Guo, Shuangyue Li, Qing Zhang, Xiuyan Sun, Fengyuan Piao

**Affiliations:** 1 Department of Occupational and Environmental Health, school of public health, Dalian Medical University, Dalian 116044, China; 2 Department of Environment Hygiene Division, Dalian Center for Disease Control and Prevention, Dalian 116021, China; 3 Department of Integrative Laboratory, Affiliated Zhongshan Hospital of Dalian University, Dalian 116001, China

**Keywords:** 2,5-Hexanedione, Neurotoxicity, ProNGF, Apoptosis, JNK pathway

This article is being retracted from *Bioscience Reports* at the request of the Editor-in-Chief and the Editorial Board.

This follows the receipt of a notification from a reader, alerting the Editorial Board to concerns over potentially incorrect apoptosis measurements in Figure 2. There are also concerns over the western blots: in Figure 5B, the GAPDH bands are thought to be presented with an incorrect molecular weight, and different orientations of loading controls compared to the rest of the blots in corresponding panels leads to concerns that the loading controls were not run on the same gel.

Similarities were also observed by the Editorial Office between the following images:

- In Figure 1B, regions of the blue and yellow boxes appear highly similar despite originating from different sections of the larger sample.
- The Figure 4B c-Jun bands appear highly similar to the Figure 7F Beta-actin bands.

The authors have been contacted with regard to the retraction and have not responded to the Journal's queries or the concerns raised.

Given the extent of the issues raised, the Editorial Board stands by the decision to retract the article.

